# Is Fentanyl Rebound an Intrinsic Feature of Naloxone Reversal?

**DOI:** 10.3390/ph18111634

**Published:** 2025-10-29

**Authors:** Michael Voronkov, Georgiy Nikonov, Melda Uzbil, George Milevich, John Abernethy, Inès Barthélémy

**Affiliations:** 1 Serodopa Therapeutics Inc., Gainesville, FL 32601, USA; 2 Alfacheminvent LLC, Alachua, FL 32615, USA; 3 CARE-NMD Platform, Ecole Nationale Vétérinaire d’Alfort, 94700 Maisons-Alfort, France

**Keywords:** fentanyl, overdose reversal, naloxone, pharmacokinetics

## Abstract

**Background/Objectives**: The drug development response to the unique pharmacology of fentanyl, which drives the current opioid epidemic, has primarily focused on increasing naloxone doses and employing longer-acting antidotes. While having lower withdrawal liability, the commonly perceived disadvantage of naloxone is its reduced effectiveness against re-narcotization or “fentanyl rebound,” due to a significant mismatch between its half-life (t_1/2_) and that of fentanyl. **Methods**: We conducted a pharmacokinetic profile (PK) crossover study in fentanyl-sedated dogs to assess naloxone (NX) and its lipophilic prodrug (NX90) with regard to fentanyl PK and re-narcotization risk. **Results**: Our findings showed that naloxone redistributed fentanyl into the plasma, with correlating (R^2^ = 0.9121) fentanyl and naloxone plasma levels when seven plasma samples per dog for each treatment (including placebo) were analyzed. This redistribution led to reductions in fentanyl’s volume of distribution at steady state (V_ss_: 11.8 ± 1.7, 8.4 ± 2.4, and 8.7 ± 2.6 L/kg), mean residence time (MRT: 19.9 ± 1.8, 18.6 ± 7.2, and 16.2 ± 8.8 min), and half-life (t_1/2_: 14.3 ± 1.9, 13.0 ± 4.9, and 11.2 ± 6.1 min) after the administration of a placebo, NX, and NX90, respectively. Additionally, we observed that the delay in the transient re-sedation (re-narcotization) of the dogs correlated (R^2^ = 0.794) with naloxone’s exposure (AUC_inf_). These data suggest that (i) the displacement of fentanyl into a metabolically active compartment and (ii) the delay in re-narcotization risk are both independent of naloxone’s half-life and are likely to be more effectively achieved with higher doses of naloxone. **Conclusions**: Combined with the lower risk of precipitating protracted withdrawal, these findings support the clinical use of higher-dose naloxone over longer-acting antidotes for reversing fentanyl-related overdoses.

## 1. Introduction

The anomalous pharmacology of fentanyl—including its rapid onset of action, decreased sensitivity to reversal by naloxone [1], increased risk of Wooden Chest Syndrome [2], and association with ventricular arrhythmias [3,4]—has contributed to a dramatic rise in synthetic opioid-related deaths. By 2022, there had been 107,081 drug overdose (OD) deaths attributed to synthetic opioids in the United States [5].

Naloxone, the gold standard for reversing opioid OD [6], requires prompt delivery, often at higher or repeated doses [7,8] in fentanyl cases, pushing the limits of the intervention safety [9]. However, quite often after a single dose of naloxone, a “fentanyl rebound” or re-narcotization is observed that is attributed to fentanyl accumulation in peripheral stores [10]. In fact, fentanyl can be detected in individuals who use illicitly manufactured fentanyl for, on average, more than 7 days [11]. Therefore, a fentanyl rebound defined as continued fentanyl exposure at the levels sufficient to exert active physiological effects is one of the major challenges for naloxone interventions.

Since naloxone’s pharmacological role is to outcompete fentanyl for the µ-opioid receptors in the brain, the displaced fentanyl as the result, according to extensive modeling [12,13], should be redistributed from the brain to the periphery. However, because the systemic elimination of naloxone is much faster than that of fentanyl, at some point, fentanyl initially pushed to the periphery by naloxone will be taken back up by the brain, potentially rendering a single administration of naloxone inefficient to fully prevent fentanyl rebound. Furthermore, if fentanyl rebound is indeed determined by a competition of antidote versus fentanyl systemic eliminations, do we really turn to antidotes with longer half-lives (t_1/2_)?

A potential flaw in this reasoning is that we do not know if by pushing fentanyl to the periphery naloxone also increases its systemic elimination. Indeed, if this were the case, then then magnitude of the fentanyl rebound would no longer be determined exclusively by the t_1/2_ of the antidote but also by its dose. However, to support this hypothesis, we could not find any definitive reports in the literature as to whether naloxone actually increases fentanyl peripheral levels. While fentanyl levels could be affected by other drugs [14,15], there is surprisingly limited data on how µ-antagonists may affect its pharmacokinetics. For instance, naltrexone appeared to have no effect on fentanyl pharmacokinetics in humans [16], and there was no correlation between the naloxone dose and fentanyl plasma concentrations in OD victims [17]. However, in samples that excluded the presence of other drugs (e.g., sedatives) fentanyl levels inversely trended with the administered naloxone equivalents, suggesting that higher doses of naloxone may have caused a higher clearance of fentanyl in OD victims.

In another more controlled clinical study, pharmacokinetic analysis did not indicate any correlations between plasma fentanyl and naloxone concentrations [18]. In fact, in about half of the patients analyzed, fentanyl levels went up with naloxone administration under the well-defined protocol, and in the rest they went down. Even though fentanyl and naloxone dosing were adjusted based on patient-specific criteria, continuous naloxone infusion appeared to correlate with the elevated plasma fentanyl levels.

We also found no conclusive animal data, yet the recent study on the pharmacodynamic interplay of fentanyl and naloxone in rats [19] offers additional clues. Measuring central and peripheral oxygenation, Kiyatkin observed that after the initial hypoxic state induced by fentanyl, there was hyperoxia in the brain and persistent peripheral hypoxia. This was attributed to the peripheral vasoconstrictive effect of fentanyl that may contribute to blood redistribution and hyperoxia in the brain. Indeed, fentanyl at higher peripheral levels has shown a robust vasoconstriction effect [20]. Therefore, the magnitude of brain hyperoxia could be indicative of changes in peripheral fentanyl levels upon naloxone intervention. Interestingly, naloxone administration within minutes also led to higher brain oxygenation, with no evidence of that in saline controls. Since naloxone has no effect on the vasoconstrictive response to hypoxia [21], that additional oxygenation would have to come from the higher peripheral fentanyl levels. Notably, the increase in brain oxygenation from naloxone was similar in magnitude to the increase caused by another fentanyl administration after another 100 min [19]. The most suggestive data come from a significant correlation between NX µ-receptor occupancy and its plasma levels in [11C]carfentanil-treated rhesus macaques, as they pose a question as to where the freed up carfentanyl went [22].

With no clear evidence of naloxone causing peripheral fentanyl levels to go up or having an effect on its systemic elimination, we conducted a pharmacokinetic assessment of fentanyl in sedated dogs treated with saline, naloxone (NX), or its prodrug (NX90) [23], which was more efficacious in the fentanyl OD model. We hypothesize that NX at higher doses might be more effective against the fentanyl rebound by redirecting fentanyl to a metabolic compartment and increasing its systemic elimination. In this report we present the first evidence of NX in a dose-dependent fashion pushing more fentanyl into the metabolically active compartment.

## 2. Results

### 2.1. Pharmacokinetics

For FT, NX, and NX90, the lower limit of quantification (LLOQ) was established at 0.5 ng/mL in plasma, and the exposure levels below the 75% of the LLOQ (0.375 ng/mL) were determined as below the limit of quantification. The fentanyl parameters were obtained after the administrations of a placebo, NX (0.12 μmol/kg), or NX90 (0.12 μmol/kg) in combination with fentanyl (10 μg/kg) and yielded respective mean plasma values of t_1/2_ at 14.3 ± 1.9, 13.0 ± 4.9, and 11.2 ± 6.1 min (min), C_0_ at 1.1 ± 0.4, 1.4 ± 0.4, and 1.2 ± 0.4 ng/mL, V_ss_ at 11.8 ± 1.7, 8.4 ± 2.4, and 8.7 ± 2.6 L/kg, and CL at 594.9 ± 134.9, 467.1 ± 67.4, and 713.7 ± 531.7 mL/min/kg (Table 1). The NX parameters were obtained after the administration of NX (0.12 μmol/kg) or NX90 (0.12 μmol/kg) in combination with fentanyl (10 μg/kg) and resulted in mean plasma values of t_1/2_ at 22.3 ± 1.4 and 21.5 ± 1.9 min, C_0_ at 80.6 ± 46.8 and 104.6 ± 60.3 ng/mL, V_ss_ at 1.9 ± 1.1 and 2.1 ± 0.8 L/kg, and CL at 103.4 ± 10.9 and 99.9 ± 33.1 mL/min/kg, respectively (Table 2). NX90 levels were close to LLOQ with C0 at 53.9 ± 80.9 and AUC_last_ at 87.2 ± 123.6 min·ng/mL.

### 2.2. Clinical Follow-Up

FT induced sedation in all the dogs and in all experiments, which was reversed within the minute following the administration of NX or NX90 (Supplementary Video S1). The dogs remained hemodynamically stable throughout the experiments, with no striking variation in arterial blood pressure. Body temperature gradually decreased during the experiments, more probably due to the immobility of the dogs monitored on an examination table. Episodes of tremors were observed in two dogs after either placebo or NX, 30 to 55 min after FT administration. Mild re-sedation or sleepiness were observed 45.0 ± 8.7 min after FT in the NX-treated animals and 51.7 ± 12.6 min after FT in the NX90-treated dogs.

### 2.3. Respiratory Monitoring

**Figure 1:**
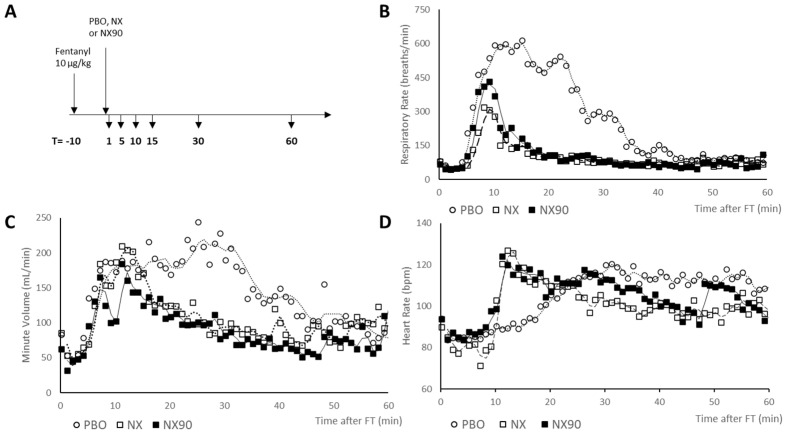
(**A**) Study design with blood samples taken at 0, 1, 5, 10, 15, 30, and 60 min. PBO—saline (iv, 0.1 mL/kg, NX—naloxone (iv, 0.12 μmol/kg), and NX90 (iv, 0.12 μmol/kg)). (**B**). Averaged (*n* = 3) respiratory rate (RR) in fentanyl-sedated dogs after saline (placebo), NX, and NX90 administration. (**C**) Averaged (*n* = 3) minute volume (MinV). (**D**) Averaged (*n* = 3) heart rate (HR); this ventricular rhythm was observed 17 min after FT administration when the placebo was given.

Five minutes after fentanyl injection, the dogs exhibited rapid shallow breathing, which was rapidly reversed by NX and NX90 in contrast to the placebo. The increased respiratory rate was associated with an increase in the minute ventilation that also responded to NX and NX90 (Figure 1C).

### 2.4. EKG Monitoring

All the dogs exhibited decreased HR after FT administration. In one of them, a marked decrease in the sinusal rhythm HR was consistently observed from around 5 min after FT injection together with the onset of a ventricular rhythm, which became the majority rhythm during the few minutes. In all the dogs, NX and NX90 induced a sudden increase in the HR towards more elevated values than at baseline, progressively decreasing thereafter. The subsequent increase in the HR was much slower after the placebo (Figure 1D). In the dog exhibiting very low HR and a ventricular rhythm after FT, NX90 completely and immediately abolished this pattern, while NX took 4 min to make the ventricular rhythm disappear (14 min after FT).

## 3. Discussion

We chose to use fentanyl-sedated dogs instead of an overdose model to avoid any complications from a potential rescue procedure during blood collection. Therefore, we used the highest fentanyl dose (10 µg/kg) recommended for inducing pre-operative analgesia in dogs [24]. The NX dose of 0.12 μmol/kg (0.04 mg/kg) was used as recommended to reverse opioid sedation in dogs [25], and NX90 was used in an equimolar dose (0.053 mg/kg). SpO2 was measured in the pre-reversion phase using a pulse oximeter and remained normal (98–99%).

**Figure 2:**
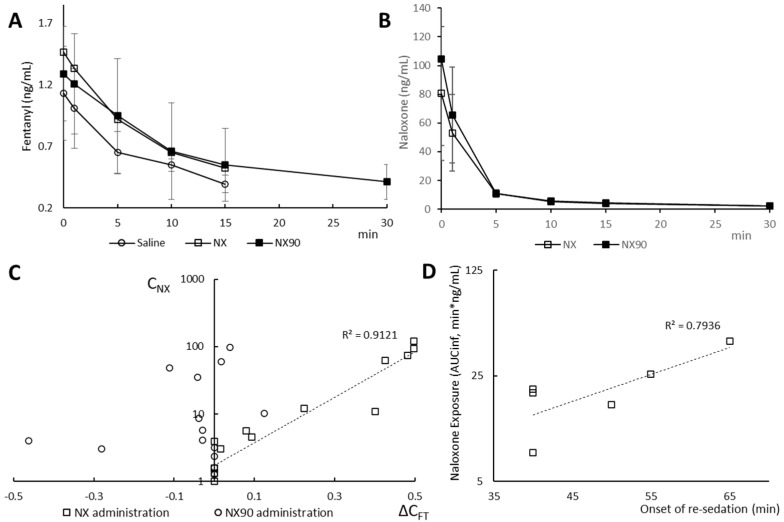
(**A**). Averaged (*n* = 3) fentanyl plasma levels after saline, NX, or NX90 administration. (**B**). Averaged (*n* = 3) naloxone plasma levels after saline, NX, or NX90 administration. (**C**). Fentanyl excess (ΔCFT) over control levels correlates with NX levels after NX administration (*n* = 14) but not after NX90 administration (*n* = 14). (**D**). Naloxone AUCinf correlates with a delay in re-sedation in NX- or NX90-treated dogs. The first dog was inadvertently administrated with saline 30 min after fentanyl; data not used.

As we hypothesized, fentanyl plasma levels upon the administration of NX or NX90 were higher than in saline-treated dogs (Figure 2A). To our knowledge, this is the first in vivo evidence of the redistribution of the free fraction of fentanyl from the brain to the blood by naloxone. It explains why after naloxone administration, a slight increase in brain oxygenation was observed by Kiyatkin [19].

Furthermore, to account for the difference in metabolic rates between animals, we analyzed the difference in FT levels (ΔC_FT_) between antidote and placebo administrations within the same dog (Figure 2C). With seven datapoints per dog analyzed, and assuming free equilibrium conditions, we found that at nearly all measurements in all experiments, higher fentanyl levels corresponded to higher naloxone levels after NX administration. Furthermore, we found an excellent correlation (R^2^ = 0.9121) between ΔC_FT_ and naloxone plasma levels. These data are complementary to previously published data on the correlation of naloxone plasma levels and freed-up opioids from µ-receptors in the brain [22]. More importantly, the observed exponential relation of excess fentanyl and naloxone plasma levels strongly suggests that higher doses of naloxone could be more effective at sequestering fentanyl to a metabolically active compartment.

The observed phenomenon is likely to be relevant and for other µ-agonists and antidotes. In fact, it could explain an uncharacteristic second peak in methadone PK profiles in dogs after intramuscular intervention with naloxone, naltrexone, or nalmefene [26]. The second methadone peak occurred just around Tmax for each antidote, consistent with the antidote’s Cmax driving higher plasma levels of methadone. This is quite remarkable given the significant differences in the t_1/2_ of naloxone, naltrexone, and nalmefene used in that study and suggests that a dose as a proxy for Cmax may be more important than antidote’s t_1/2_ in driving µ-agonists into the metabolically active compartment.

In contrast, no correlation was found between the excess of fentanyl and circulating naloxone after NX90 administration. This was unexpected, since NX90 rapidly converts to NX in vivo. One explanation is that NX90 has lipophilicity (defined as a cLogD at physiologically relevant pH) that is about 30 times higher than that of NX [23] and similar to fentanyl, making it more likely to be distributed to the same peripheral stores as fentanyl. Indeed, displacing fentanyl simultaneously from the brain and peripheral stores is consistent with a higher fentanyl clearance in NX90-treated animals (Table 1). The higher FT systemic clearance should also effectively mitigate fentanyl-related toxicities (e.g., abnormal ventricular rhythms or renarcotization).

Accordingly, we observed no tremors or ventricular rhythms in NX90-treated animals that were noted in saline and some NX-treated dogs. This also could be the explanation for why we previously did not observe renarcotization in an FT overdose rat model with NX90 [23].

While the redistribution is clearly taking place, fentanyl is being distributed to peripheral stores to a lesser degree than in the saline group, as V_ss_ is reduced by 28% after NX and by 26% after NX90 administration (Table 1). This means that more fentanyl freed up from brain is staying in the metabolically active compartment. As a result, the mean residence time (MRT) of fentanyl in the body is reduced and the chances of fentanyl rebound should go down. This might be in contrast to “peripheral sink” antidotes such as fentanyl-specific mAb [27,28] and other sequestrants [29]. Indeed, while fentanyl is sequestered away from the brain, it is also sequestered away from the systemic elimination, potentially increasing the MRT of fentanyl in the body. However, there are no published data of fentanyl’s fate in a “peripheral sink” approach and whether the increased fentanyl MRT would result in adverse peripheral or CNS effects.

However, do higher plasma levels (e.g., higher doses) of NX influence fentanyl rebound? In this study, NX-treated animals became briefly sleepy again at 45.0 ± 8.7 min and NX90-treated animals did so in 51.7 ± 12.6 min after FT injection, well after its plasma levels dropped below 0.95 ng/mL, the threshold associated with CNS effects in dogs [30]. To account for inter-animal differences in the metabolic rate, we compared individual naloxone exposures (NX AUC_INF_) with re-sedation times in each dog as a measure of potential fentanyl rebound (Figure 2D). The onset of this rebound correlated (R^2^ = 0.794) with NX AUC_INF_, suggesting that the more fentanyl that is displaced into a metabolically active compartment, the longer it may take for it to redistribute and potentially cause re-sedation. One could suggest that with a certain delay, there might not be enough fentanyl left in the body to cause secondary intoxication. This finding is unexpected, as it challenges the commonly held assumption that the antidote’s half-life, rather than systemic exposure, is the critical factor in preventing fentanyl rebound.

Our observation aligns with a recent study [26] reporting an even more remarkable lack of re-sedation (e.g., methadone rebound) in dogs after naloxone administration, despite naloxone having a much shorter half-life than alternatives like naltrexone or nalmefene. These findings further support the idea that fentanyl rebound risk is more dependent on the antidote’s systemic exposure (i.e., dose and AUC) than on its elimination half-life.

Study limitations: The study was conducted with a low dose of fentanyl which was not enough to cause an overdose in dogs. Therefore, the interpretation of the data has limited significance for fentanyl overdose. Another limitation is that a typical number (*n*= 3) of animals used for a PK profile evaluation is low for the statistical analysis. Thus, significance in FT PK parameters was reached (two-tailed *t*-test) only for NX90-treated animals (t_1/2_, MRT and Vss) when compared to placebo-treated animals. Our study has not evaluated NX accumulation in compartments other than plasma, which further limits the interpretation of Vss.

Clinical relevance: Overall, our data suggest that fentanyl rebound is determined not so much by the fast systemic elimination of naloxone and therefore by its t_1/2_ but by its circulating levels and therefore by its dose. As fentanyl exposure becomes increasingly common, this finding has important clinical implications: rather than relying on reversal agents with a longer t_1/2_, the use of higher-dose naloxone boluses may achieve more sustained reversal without necessarily increasing the risk of protracted withdrawal in patients with OUD.

## 4. Materials and Methods

### 4.1. Chemicals

Acetonitrile (ACN; J.T.Baker^®^, Phillipsburg, NJ, USA), Dimethyl sulfoxide (DMSO; Honeywell Research Chemicals, Muskegon, MI, USA), Formic acid (FA; Merck, Darmstadt, Germany), and Oxybutynin (Sigma, St. Louis, MO, USA) were used. FT (Fentadon^®^, batch #143976 exp 06-2025 Dechra, Northwich, UK) and NX (Narcan^®^, SERB, batch #3087, exp 09-2024, Paris, France) were purchased from commercial sources. NX90 (batch # NX-90-1-114-1) was supplied by Alfacheminvent, LLC (Alachua, FL, USA). NX and NX90 were used as HCl salts.

### 4.2. Animals

The selection of the species for this study was dictated by a need to evaluate the PK parameters of NX90 in dog since NX90 stability in human plasma was closer to dogs’ plasma stability rather than to rats’ plasma stability. Three young adult (two females, one male) beagle dogs were included in the study, aged 12.8, 13.4, and 13.6 months at the first experiment. The animal experimentation adheres to the declaration of transparency and scientific rigor [31]. All animal experiments were performed in accordance with relevant guidelines and regulations, were approved by the Ethical Committee of EnvA, ANSES, and UPEC, and were authorized (#41958-2023032211493311v2) by the French Ministry of Research. No anesthetic agent was used; the experiments were performed on conscious dogs. The dogs were not euthanized at the end of the experiments and were rehomed.

### 4.3. Study Design

The study was designed as a cross-over study. Each of the three dogs underwent three experiments during which they received fentanyl followed by a reversal with either NX, NX90, or a placebo (Figure 2A). A one-week wash-out was planned between each experiment, and the order of the reversal agent tested in the three experiments was randomly pre-established and varied for each dog. Doses for fentanyl and naloxone were selected per published protocol [32]. The dogs were habituated to the inductive belts/jacket required for monitoring respiratory function, ECG, and blood pressure. It is worth noting that the device used for this wireless monitoring (EmkaPACK 4G system, Emka technologies, Paris, France) is adapted to dogs, and in our experience, it is very well tolerated.

### 4.4. Pharmacokinetic Study

One week before the pharmacokinetic (PK) study, an arterial catheter (Venocan 24 G) was inserted into one of the dorsal pedal arteries (pelvic limb, foot). One milliliter blood samples were taken (the amount of blood taken from each animal never exceeded 0.5% of the total volume) at the arterial catheter after withdrawing the blood already present in the catheter, just before and at the following timepoints after the reversal agent injection: 1, 5, 10, 15, 30, and 60 min. The catheter was then rinsed using heparinized NaCl 0.9% through the arterial line. The samples were immediately transferred to EDTA-tubes on wet ice. They were then centrifuged at 4 °C and 3000 rpm for 5 min within a maximum of 15 min. Plasma was divided into a 250 µL aliquot and another aliquot containing the remaining amount of plasma. Cryovials were then transferred and stored at −80 °C.

The determination of the exposure levels of NX, NX90, and fentanyl in plasma samples, collected from canines, was performed by liquid chromatography–tandem mass spectrometry (LC-MS/MS, Triple Quad™ 5500, Sciex, Inc., Framingham, MA, USA; Agilent Poroshell 120 EC-C18 column, 2.7 μm, 3.0 × 50 mm, Agilent Technologies, Inc., Santa Clara, CA, USA). The work was performed by Pharmacology Discovery Services Taiwan, Ltd. The lower limit of quantification (LLOQ) was 0.5 ng/mL in plasma. The PK parameters (e.g., elimination half-life (t_1/2_), maximim concentration (C0), measured exposure (AUC_last_), mean residence time (MRT), volume of distribution at steady state (V_ss_), and clearance (CL)) of NX, NX90, and fentanyl after the IV administrations of placebo, NX, or NX90 in combination with fentanyl were obtained from the non-compartmental analysis (NCA) of the plasma data using WinNonlin 8.3.5.

### 4.5. Experimental Procedure

Respiratory monitoring: Respiratory inductance plethysmography (RIP) was performed by placing inductive belts around the thorax and abdomen and maintaining them in place thanks to a jacket in which they were inserted. Wires were connected to an emitter (EmkaPACK 4G, Emka technologies, Paris, France). The signal of the belt stretching was acquired at 100 Hz during Tidal breathing in the software IOX v.2.9.5.73 (Emka technologies). The signal was calibrated by a concomitant spirometric recording during a few respiratory cycles, obtained through a pneumotachometer linked to a facemask (EmkaPACK 4G system, Emka technologies).

EGC monitoring: Four ECG electrodes were clipped on four cutaneous patches placed at standardized positions in lateral recumbency: an LA electrode was positioned at the level of the apex beat, and RA was placed symmetrically on the right side; LL and RL electrodes were respectively positioned in the alignment of LA and RA, at the level of the umbilicus. Electrodes were connected to the same emitter as the one used for respiratory monitoring, which was then placed into a dedicated jacket worn by the dog. Six lead (I, II, III, aVL, aVF, aVR) ECGs were acquired at a sampling rate of 500 Hz in the software IOX v.2.9.5.73 (Emka technologies), concomitantly with the RIP traces. Both respiratory and ECG signals were acquired continuously from 30 min before fentanyl injection until 5 h after the injection of the reversal agent.

Arterial pressure monitoring: Non-invasive arterial pressure monitoring was also performed (PetMAP Graphic II, Ramsey Medical), as well as rectal temperature monitoring (Genia, 0.1 °C accuracy), and the result was noted every 10 min during 30 min before fentanyl injection until 1 h after the injection of the reversal agent.

Fentanyl Injection: Thirty minutes after the beginning of the follow-up, Fentanyl (Fentadon^®^, Dechra, batch #143976 exp 06-2025) at 10 µg/kg (i.e., 0.2 mL/kg) was injected through a venous catheter over one minute. This dose corresponds to the highest dose recommended in the market approval for inducing per-operative analgesia in dogs. At the end of the injection, 2 mL NaCl 0.9% were injected to rinse the catheter.

Reversal agent injection: Ten minutes after the initiation of fentanyl injection, the reversal agent was administered through a rapid IV bolus, followed by 2 mL NaCl 0.9% to rinse the catheter. The volume was kept at 0.1 mL/kg, whatever the agent tested. NX (Narcan^®^, SERB, batch #3087, exp 09-2024) was injected at a dose of 0.04 mg/kg (0.12 μmol/kg), the dose recommended in dogs to reverse opioids. NX90 (batch # NX-90-1-114-1) was injected at a dose of 0.053 mg/kg (0.12 μmol/kg, 1.3× correction factor, taking into account differences in molecular weight). The placebo (NaCl 0.9%) was injected at a dose of 0.1 mL/kg.

### 4.6. Data Analysis

Respiratory traces analysis: RIP trace analysis was performed in the software ecgAUTO v3.3.0.4 (Emka Technologies) after having calibrated the belts signals from the pneumotachometer measurement. The calibration coefficient obtained was then used to analyze all three traces from each dog. An automated analysis was run after having determined adequate settings to allow for proper respiratory cycles detection. Each respiratory cycle was analyzed, and the following parameters were studied: respiratory rate (RR, bpm), Tidal volume (mL), and Minute volume (mL/min).

ECG analysis: Quantitative ECG analysis was performed in the software ecgAUTO v3.3.0.4 (Emka technologies). Automated RR detection was run on each trace, on the lead allowing for the best RR detection (lead I or lead II). For each beat, the heart rate (HR) was calculated. The heart rate was then averaged over 1 min periods.

### 4.7. Statistical Analysis

The order of the reversal agent tested among the three experiments was randomly pre-established and was different for each dog. The key conclusion is based on analyses of 14 data points of fentanyl and naloxone plasma levels. The data were analyzed using a two-tailed *t*-test. The statistical significance level was set at α < 0.05.

## 5. Conclusions

First in vivo evidence of naloxone-driven fentanyl redistribution

This study provides the first in vivo evidence of naloxone redistributing the free fraction of fentanyl from the brain to plasma.

Impact on Fentanyl Rebound

The naloxone-driven redistribution of fentanyl, combined with the reduced volume of distribution and shorter mean residence time, suggests that the risk of fentanyl rebound—where fentanyl re-enters the central nervous system—is likely reduced in a dose-dependent fashion. Therefore, higher doses of NX are likely to be more effective against fentanyl rebound by pushing more fentanyl into the metabolically active compartment without tying up its free fraction.

Reduced Fentanyl Toxicity with NX90

No tremors or ventricular arrhythmias were observed in NX90-treated animals, in contrast to those receiving fentanyl alone or NX-treated animals. At the doses used in the study, the fentanyl rebound was delayed proportionately to the fentanyl displaced by an antidote from the brain.

Future directions

A larger dose response study is needed to demonstrate the further mitigation of fentanyl rebound by NX and NX90. Another direction is to explore the effects of the emerging class of various naloxone potentiators [33,34,35] in fentanyl overdose on fentanyl pharmacokinetics.

## Figures and Tables

**Table 1:** The PK parameters of FT in canine plasma samples after saline, NX, or NX90 administration.

Intervention	t_1/2_	C_0_	AUCl_INF_	MRT	V_ss_	CL
	(min)	(ng/mL)	(min·ng/mL)	(min)	(L/kg)	(mL/min/kg)
NaCl (0.9%, IV)	14.3 ± 1.9	1.1 ± 0.4	17.3 ± 3.9	19.9 ± 1.8	11.8 ± 1.7	594.9 ± 134.9
NX (0.12 µmol/kg, IV)	13.0 ± 4.9	1.4 ± 0.4	21.7 ± 3.4	18.6 ± 7.2	8.4 ± 2.4	467.1 ± 67.4
NX90 (0.12 µmol/kg, IV)	11.2 ± 6.1	1.2 ± 0.4	22.1 ± 18.1	16.2 ± 8.8	8.7 ± 2.6	713.7 ± 531.7

**Table 2:** The PK parameters of NX in canine plasma samples after NX or NX90 administration.

Intervention	t_1/2_	C_0_	AUC_INF_	MRT	V_ss_	CL
	(min)	(ng/mL)	(min·ng/mL)	(min)	(L/kg)	(mL/min/kg)
NX (0.12 µmol/kg, IV)	22.3 ± 1.4	80.6 ± 46.8	389.4 ± 38.9	18.0 ± 8.1	1.9 ± 1.1	103.4 ± 10.9
NX90 (0.12 µmol/kg, IV)	21.5 ± 1.9	104.6 ± 60.3	439.3 ± 177.7	16.0 ± 2.3	2.1 ± 0.8	132.5 ± 44.1

## Data Availability

The raw data supporting the conclusions of this article will be made available by the authors on request.

## Supplementary Materials

The following supporting information can be downloaded at: https://www.mdpi.com/article/10.3390/ph18111634/s1, Video S1: Tapioca_NX90_recovery.
